# Editorial: Nephrotic Syndrome in Children

**DOI:** 10.3389/fped.2021.803923

**Published:** 2021-11-24

**Authors:** Sami A. Sanjad, Tim Ulinski, Bilal Aoun

**Affiliations:** ^1^Department of Pediatrics/Nephrology, American University of Beirut Med Center, Beirut, Lebanon; ^2^Department of Pediatric Nephrology, Armand Trousseau Hospital, Paris, France

**Keywords:** nephrotic syndrome, corticosteroids, immunosuppressives, rituximab, mycophenolate mofetil, interleukins, NPHS1, NPHS2

Although rare in absolute terms, with an incidence of 2–7/100,000 children/year (1), the nephrotic syndrome is the most common glomerular disease in childhood. As a clinicopathological entity, it is characterized by abnormal glomerular basement membrane permeability to plasma proteins (mainly albumin) resulting in massive proteinuria (albuminuria), and varying degrees of hypoalbuminemia edema and hyperlipidemia. Seventy to 80% of children with the nephrotic syndrome attain complete remission with various corticosteroid regimens and are labeled as steroid sensitive. They have no abnormalities detected on kidney biopsy by light microcopy, hence the term minimal change disease. Unfortunately, two out of three steroid responders have at least one relapse within the first 6 months from stopping treatment (2).

The current issue of Frontiers in Pediatric Nephrology is dedicated to the nephrotic syndrome. Fourteen articles, including 5 case reports, address various aspects of this disease. Of these, four, including a case report, are devoted to different therapeutic approaches to the nephrotic syndrome.

In a multicenter prospective cohort study from Italy, Pasini et al. demonstrated that time to remission in children receiving different steroids regimens had no effect on the relapse rate. They found, however, that younger age and low total serum protein were independent predictors of relapse risk. The controversy over the treatment with corticosteroids and whether the cumulative dose or the duration of therapy may affect the relapse rate is not fully resolved and the jury is still out (1, 3). Two meta-analyses from China, one addressing immunosuppressive therapy with rituximab, Gao et al. the other with mycophenolate mofetil, Xiang et al. for steroid dependent (SDNS) and frequent relapsing nephrotic syndrome (FRNS) attest to the efficacy and safety of these agents in reducing the number of relapses in addition to their steroid sparing effect. The first reports about benefits of rituximab in nephrotic syndrome were published in the early 2000's and consisted of case reports and small case series ranging from 1 to 24 patients with SDNS or FRNS (4). In addition to its immunological role, Rituximab may have direct and non-immunological effects on podocytes associated with inducing remission of proteinuria in children and adults with FSGS (5). Mycophenolate mofetil has been described as a promising drug in SDNS, mainly in maintaining long-term remission. This drug seems to be well-tolerated in most children and its efficacy in maintaining remission in SDNS and FRNS has been demonstrated, especially when compared to other immunosuppressives that might be nephrotoxic and require frequent monitoring of blood levels (6).

Lastly, Ma et al. from China report the case of a 7-year-old girl with SDNS due to C1q nephropathy who went into complete remission with a low dose of rituximab (93 mg/m^2^). This is one fourth of the usual dose used in patients with SDNS and FRNS. The authors raise the issue of reconsidering the dose in this disease and we believe it may be worth trying such a dose in a controlled manner in those patients as well. This might help avoid both short- and long-term side effects of rituximab.

Five papers address molecular and genetic aspects of the nephrotic syndrome:

In a study by Bai et al. a novel missense mutation on the NPHS2 gene was found in Chinese children with steroid-resistant nephrotic syndrome. This gene plays a significant role in proper podocin protein function and its pathogenesis (7). Thus, we encourage pediatric nephrologists to perform genetic testing on all patients with SRNS, especially those with early-onset SRNS and a positive family history as it helps in avoiding unnecessary immunosuppressive and potentially toxic medications. Looking to other possible genetic mutations that play a role in patients with SRNS. Liu et al. from China, identified a novel, likely pathogenic mutation in the CD2AP gene. This is a crucial protein for slit-diaphragm assembly and function (8). Again, such new mutations ought to lead to multicenter, international collaborative studies that might help identify other pathogenic variants in patients with SRNS. Jacob et al. from the United Arab Emirates report the case of a male with congenital nephrotic syndrome (CNS) associated CMV infection but no syndromic stigmata or systemic manifestations. Kidney biopsy showed crescents, fibrosis and tubulointerstitial changes, but was negative for CMV inclusion by EM. Genetic studies revealed a novel homozygous mutation in the *NPHS1* gene, highlighting the importance of genetic testing all patients with CNS.

Shah et al. report the case of 15-year-old girl who developed acute onset nephrotic syndrome with biopsy proven membranous nephropathy in association with repeated use of the NSAD ibuprofen for dysmenorrhea. While NSAIDs are a common cause of acute interstitial nephritis and AKI with nephrotic range proteinuria (9) the case reported here was that of a pure membranous nephropathy, a rare complication of NSAID therapy indeed.

In the era of B cell depleting agents for the management of steroid dependent or steroid resistant forms of idiopathic nephrotic syndrome (INS) it is obvious that B cells are believed to have major impact in its pathophysiology. However, research also focuses on interleukins and Al Rushood et al. have searched for polymorphisms in the IL4 and IL13 gene. Only the IL13 RQ genotype polymorphisms have been found more often in steroid sensitive patients compared to steroid resistant ones, but no IL polymorphism could be identified as susceptibility factor for INS compared to controls.

The remaining five papers deal with different and interesting aspects of the nephrotic syndrome, including metabolic and infectious complications and the role of T cell cytokines in the pathogenesis of nephrotic syndrome.

Turolo et al. report from Milan, Italy report on the persistent elevation of the omega-6 fatty acids, arachidonic acid and its precursor linoleic acid in nephrotic children during remission, while free of proteinuria. The authors go on to suggest that these fatty acid levels might be regarded as candidate biomarkers for the risk of relapse in children with nephrotic syndrome. A somewhat parallel observation was reported many years ago by Zilleruelo et al. (10) in children with nephrotic syndrome who showed elevated total cholesterol, triglycerides, LDL and VLDL during remission.

Another interesting aspect concerning T cell function has been examined by Ni et al. T Helper type 2 (Th2) cells secrete various cytokines such as IL4 and IL13 which trigger IgE secretion. High levels of IgE in patients with active INS are believed to be due to these changes in Th2 cell function. The authors showed, that micro-RNAs seem to play a role for Th2 expression and in children with active non-atopic INS, the levels of miRNA-24 and-27 are decreased, allowing a higher number of Th2 cells and a Th1/Th2 imbalance.

Infections are a common cause of morbidity, and before the era of antibiotics, the highest cause of mortality in children with nephrotic syndrome (11). Zhang et al. from China investigated the risk factors in a large number of children with nephrotic syndrome. As expected, steroid resistance combined with the use of immunosuppressive drugs were significant risk factors for the development of severe infection. Gram positive bacteria, elevated CRP above 8 mg/L and low C3 complement level (<0.55 gm/L) and a low absolute lymphocyte count (<1.5 × 10^9^/L) were major risk factors for severe infection and mortality.

As pediatric patients with INS are at risk for infections which may be preventable by specific vaccinations, there is a strong need for clear information from the caregivers to the patients' families. Vaccination strategies (in particular anti-pneumococcal vaccination) have improved tremendously and the number of invasive pneumococcal infections in patients with active INS has decreased. Tran et al. assessed parental and pediatric nephrologist knowledge about immunization practices in children with nephrotic syndrome. Surprisingly only 44% of pediatric nephrologists adhered to the Advisory Committee on Immunization Practices (ACIP) guidelines for inactive vaccines and only 22% for live vaccines.

Congenital nephrotic syndrome is a severe clinical entity with several consequences for the patients and their families. As profound hypoalbuminemia increased the risk for developmental problems, in particular under the age of 3 years, strategies to correct at least partially the serum albumin level have to be established, but these often require long term hospitalization. The work of Eugenia Serramontmany et al. showed that in those patients who require only one daily albumin infusion, home albumin infusion therapy is a reasonable option and does not increase the risk of complications such as central venous catheter infections.

Idiopathic nephrotic syndrome remains enigmatic in many ways, whether from the etiologic, pathophysiologic or therapeutic standpoints. Prevention of related complications is still a matter of debate and many strategies deserve to be explored. The decision, when to use which immunosuppression and for what patient will remain debatable until the precise pathophysiology of INS is elucidated.

## Author Contributions

All authors contributed in a significant capacity to this editorial on Nephrotic syndrome in children.

## Conflict of Interest

The authors declare that the research was conducted in the absence of any commercial or financial relationships that could be construed as a potential conflict of interest.

## Publisher's Note

All claims expressed in this article are solely those of the authors and do not necessarily represent those of their affiliated organizations, or those of the publisher, the editors and the reviewers. Any product that may be evaluated in this article, or claim that may be made by its manufacturer, is not guaranteed or endorsed by the publisher.
